# Perioperative outcome and complications following single-staged Posterior Spinal Fusion (PSF) using pedicle screw instrumentation in Adolescent Idiopathic Scoliosis (AIS): a review of 1057 cases from a single centre

**DOI:** 10.1186/s12891-021-04225-5

**Published:** 2021-05-04

**Authors:** Mun Keong Kwan, Kwong Weng Loh, Weng Hong Chung, Chee Kidd Chiu, Mohd Shahnaz Hasan, Chris Yin Wei Chan

**Affiliations:** 1 Department of Orthopaedic Surgery (NOCERAL), Faculty of Medicine, University of Malaya, 50603 Kuala Lumpur, Malaysia; 2 Department of Anaesthesiology, Faculty of Medicine, University of Malaya, 50603 Kuala Lumpur, Malaysia

**Keywords:** Adolescent idiopathic scoliosis, Perioperative outcome, Complication, Single-staged, Posterior spinal fusion, Pedicle screw

## Abstract

**Background:**

There has been a growing interest in using all pedicle screw construct in posterior spinal fusion (PSF) for adolescent idiopathic scoliosis (AIS) surgery in recent years. However, studies focusing on perioperative outcome and complications utilizing only pedicle screw system in AIS population are lacking. This study aims to evaluate perioperative outcomes and to determine the prevalence of major and minor complications following single-staged PSF for AIS.

**Methods:**

In this retrospective study of prospectively collected data, 1057 AIS patients operated between 2012 and 2019 were included. Main outcome measures were operative time, intraoperative blood loss, allogeneic blood transfusion rate, length of hospital stay after surgery, complication rate, and mean drop of haemoglobin (Hb) level. We documented the number of fusion levels, screw density, and postoperative radiographic parameters.

**Results:**

There were 917 females and 140 males. Majority were Lenke 1 curve type (46.9%). Mean age was 15.6 ± 3.7 years, with mean BMI of 18.6 ± 3.2 kg/m^2^. Mean operative time was 146.8 ± 49.4 min. Average intraoperative blood loss was 952.9 ± 530.4 ml with allogeneic blood transfusion rate of 5%. Mean screw density was 1.27 ± 0.21 screws per fusion level. Average hospital stay after surgery was 3.5 ± 0.9 days. Twenty-four complications were documented: twelve superficial infections (1.14%), five transient neurological deficits (0.47%), two deep infections (0.19%), two superior mesenteric artery syndrome, and one case each (0.09%) for massive intraoperative blood loss, intraoperative seizure, and lung atelectasis.

**Conclusion:**

AIS patients treated with single-staged PSF using pedicle screw construct had a 0.95% rate of major complications and 1.32% rate of minor complications. Rate of neurologic complication was 0.47% while non-neurologic postoperative complications was 1.80% with infection being the leading complication at 1.32%.

## Background

The overall complication rate for corrective surgeries in adolescent idiopathic scoliosis (AIS) patients was 6.3% with overall mortality rate of 0.014 [1] to 0.02% [2]. These perioperative complications include intraoperative blood loss, neurological deficit, infection, vision loss and death [3], with excessive blood loss being one of the commonest as reported by Carreon et al. [4] Risks of perioperative complications such as massive haemorrhage requiring use of allogeneic blood transfusion were higher in patients with severe scoliosis [5, 6], prolonged duration of surgery and usage of combined anterior-posterior approach or when spinal osteotomies were performed [7–9]. The prevalence of neurologic complications ranged from 0.3 to 2.6% [10, 11], whereas non-neurologic complications ranged from 0 to 7.7% [12, 13]. The overall complication rate for posterior spinal fusion (PSF) ranged from 1.4 to 5.2% [13, 14]. Most studies had heterogenous study population with inclusion of patients who underwent anterior and posterior spinal surgeries; patients who underwent different instrumentation techniques (rods, hooks or screws) and patients who were diagnosed as idiopathic and non-idiopathic scoliosis [4, 15–18].

In recent years, there has been a growing interest in using all pedicle screw construct in PSF for AIS surgery [19, 20]. However, few studies have focused on perioperative complications utilizing only pedicle screw system in the paediatric population, and even fewer, on AIS patients only [21–25]. Therefore, the aim of this study was to evaluate perioperative outcomes and to determine the prevalence of major and minor complications following single-staged PSF for AIS.

## Materials and methods

This was a retrospective study of prospectively collected data involving AIS patients who underwent single-staged PSF in a tertiary academic centre between January 2012 and December 2019. This study was approved by our institutional ethical board (MREC: 2018913–6677). The inclusion criteria were all AIS patients (all Lenke types) who underwent PSF surgery between the ages of 10 and 30 years old. From the proportion of patients who were operated at the age > 18 years old, the diagnosis of idiopathic scoliosis was made during the adolescent age. The exclusion criteria were early-onset scoliosis, non-idiopathic scoliosis, patients with incomplete data and patients who underwent revision surgery or anterior surgery. Operated patients whose diagnosis of idiopathic scoliosis was made after the age of 18 were excluded in our study.

Demographic data that were collected included age, gender, height, weight, body mass index (BMI), total body surface area and Lenke curve types [26]. Intraoperative and perioperative data such as number of fusion levels, number of screws, screw density, preoperative and postoperative radiographic parameters, operative time, intraoperative blood loss, preoperative and postoperative haemoglobin (Hb) level, use of allogeneic blood transfusion, length of hospital stay after surgery and complication rate were also documented.

In our study, total intraoperative blood loss was estimated from the cell salvage system using the formula:

$$ Total\ blood\ loss\ (ml)=\left( Final\ volume\ accumulated\ in\ the\ reservoir\right)-\left( Total\ volume\ of\ anticoagulant\ citrate\ dextrose\ \left[ ACD\right]\right)-\left( Total\ fluid\ used\ for\ irrigation\ intraoperatively\right)+\left( Total\ unfiltered\ blood\right) $$

The total volume of ACD and unfiltered blood (difference between weights of used and dry reservoir) was calculated. Total irrigation fluid was measured intraoperatively. Massive blood loss was defined when total blood loss > estimated body blood volume (calculated based on Nadler’s formula) [27]. All major and minor complications were reviewed and categorized according to severity as proposed by Harms Study Group [28]. A complication was defined as major if the patient required reoperation, was considered life-threatening, or resulted in spinal cord or nerve root injury. Any minor complication could be reclassified as a major complication if it resulted in prolonged hospitalization of > 2 days, required re-admittance, or required another surgery [28]. Complications were defined as “perioperative” if they occurred within 6 weeks following surgery.

### Surgical technique

All surgeries were performed by either two spine consultants, or a combination of one spine consultant and an orthopaedic surgeon undergoing spine surgical training. Intravenous tranexamic acid was administered to all patients prior to skin incision. Controlled hypotensive anaesthesia was maintained during exposure. Patients were kept normothermic throughout the surgery. Spinal cord monitoring was used during the surgery. Three to four screws were inserted as proximal and base anchors. Alternate level screw placement was performed in between the proximal and base anchors. Correction manoeuvre consisted of rod translation and direct vertebral rotation. Facetectomies were performed to increase the spinal flexibility prior to correction as well as to assist the fusion process. Autogenous bone graft was obtained from the spinous processes, laminae, facet joints and transverse processes were processed and distributed over the fusion bed to augment the fusion process [29]. No allograft or bone substitutes were used. A subfascial drain was inserted prior to closure. Subcutaneous bupivacaine was infiltrated before skin closure.

### Postoperative protocol

Intravenous morphine delivered through patient-controlled analgesia (PCA) was provided for a minimum of 24 h after surgery and was discontinued once consumption falls below 5 mg/24 h. Oral celecoxib (Celebrex) 200 mg once or twice daily and acetaminophen tablets 500 to 1000 mg 6 hourly were started once patients could tolerate orally. Oxycodone hydrochloride (Oxynorm) capsule 5 mg was used to manage breakthrough pain. Postoperative haemoglobin (Hb) level was checked at 48 h after surgery. Allogenic blood transfusion threshold was Hb level below 8 g/dL or when patient has symptomatic anemia. The suction drain was clamped until 18 to 24 h after surgery. Upon release, a maximum of 200 ml was drained and the drain was subsequently removed. Postoperative drainage was not included in the measurement of blood loss. Accelerated recovery protocol was practiced [30].

### Statistical analysis

All data were stored and analysed using the SPSS v 23.0 (IBM Corp., Armonk, NY, USA). Categorical variables were expressed in number (n) and percentage (%) and continuous variables were expressed in mean ± standard deviation (SD). Year-over-year analysis was done using one-way analysis of variance (ANOVA) to look for significant change in the trend of mean correction rate, mean duration of surgery and mean intraoperative blood loss. All reported *P*-values were two-tailed and the cut-off point of statistical significance was 0.05.

## Results

One-thousand one-hundred and forty-six AIS patients underwent PSF from January 2012 to December 2019. One thousand fifty-seven patients fulfilled the inclusion criteria and were included in the study. Eight hundred eighty-three patients (83.5%) were aged ≤18 years before operation. Nine hundred seventeen patients were female (86.8%). The average age of patients was 15.6 ± 3.7 years old. The mean height was 157.0 ± 7.8 cm with the mean weight of 46.0 ± 9.0 kg. The body mass index (BMI) distribution was positively skewed with most patients having a low-to-normal BMI (mean BMI of 18.6 ± 3.2 kg/m^2^), and a long tail of patients with greater BMI. The average total body surface area was 1.4 ± 0.2 m^2^. Majority were Lenke 1 curve type (46.9%). The average preoperative serum Hb level was 13.7 ± 1.2 g/dL. Majority of our patients were at least Risser 4 (713 patients, 67.5%) (Table 1).

**Table 1:** Demographic Characteristics and Operative Data

Demographics	Age 10–18	Age 19–30	Total	***p***-value
Sex, n (%)				
Male	110 (10.4)	30 (2.8)	140 (13.2)	0.09
Female	773 (73.1)	144 (13.6)	917 (86.8)	
Age (year)	14.3 ± 1.9	22.5 ± 3.2	15.6 ± 3.7	< 0.01
Menarche age (year)	12.3 ± 1.2	12.8 ± 1.2	12.4 ± 1.2	< 0.01
Distribution by Lenke type, n (%)				
1	413 (39.0)	83 (7.9)	496 (46.9)	0.48
2	189 (17.9)	38 (3.6)	227 (21.5)	
3	23 (2.2)	8 (0.8)	31 (2.9)	
4	27 (2.6)	3 (0.3)	30 (2.8)	
5	159 (15.0)	25 (2.4)	184 (17.4)	
6	72 (6.8)	17 (1.6)	89 (8.4)	
Height (cm)	156.4 ± 7.7	160.0 ± 8.1	157.0 ± 7.8	< 0.01
Weight (kg)	45.2 ± 8.8	50.0 ± 9.1	46.0 ± 9.0	< 0.01
Body mass index (kg/m^2^)	18.4 ± 3.1	19.5 ± 3.4	18.6 ± 3.2	< 0.01
Body surface area (m^2^)	1.4 ± 0.2	1.5 ± 0.2	1.4 ± 0.2	< 0.01
Preoperative haemoglobin (g/dL)	13.7 ± 1.2	13.6 ± 1.4	13.7 ± 1.2	0.10
Preoperative major curve Cobb angle (°)	66.7 ± 17.3	65.4 ± 16.5	66.5 ± 17.1	0.36
Risser sign, n (%)	3.3 ± 1.5	4.9 ± 0.6	3.6 ± 1.5	< 0.01
0	73 (8.3)	0	73 (6.9)	
1	49 (5.5)	0	49 (4.6)	
2	120 (13.6)	0	120 (11.4)	
3	102 (11.6)	0	102 (9.6)	
4	395 (44.7)	12 (6.9)	407 (38.5)	
5	144 (16.3)	162 (93.1)	306 (28.9)	
**Operative Data**				
Number of screws	14.1 ± 2.3	14.3 ± 2.4	14.1 ± 2.4	0.33
Number of levels fused	10.3 ± 2.2	10.3 ± 2.1	10.3 ± 2.2	0.84
Proximal level of fusion, median	T3	T3	T3	–
Distal level of fusion, median	L3	L2	L2	–
Screw density (screws/vertebral level)	1.27 ± 0.20	1.29 ± 0.24	1.27 ± 0.21	0.20
Duration of surgery (min)	143.9 ± 48.4	161.1 ± 52.0	146.8 ± 49.4	< 0.01
Postoperative haemoglobin (g/dL)	10.9 ± 1.5	10.9 ± 1.6	10.9 ± 1.5	0.96
Total blood loss (mL)	945.4 ± 535.7	990.5 ± 502.5	952.9 ± 530.4	0.31
Estimated blood volume (mL)	3103.0 ± 499.7	3376.1 ± 539.0	3148.0 ± 516.1	< 0.01
Total blood loss/estimated blood volume (%)	30.8 ± 17.4	29.9 ± 15.9	30.7 ± 17.1	0.51
Allogeneic blood transfusion n (%)	46 (4.3)	7 (0.7)	53 (5.0)	0.51
Length of stay (days)	3.4 ± 0.9	3.6 ± 1.2	3.5 ± 0.9	0.12
Estimated blood salvage (ml)	485.6 ± 305.6	505.5 ± 297.7	488.9 ± 304.3	0.43

The mean preoperative and postoperative major curve angles were summarized in Table 2. For thoracic curve, the mean preoperative and postoperative major curve angles were 67.9 ± 17.8° and 24.0 ± 12.1°, respectively, with an average correction rate of 65.3 ± 11.7%. The mean preoperative major curve angle for thoracolumbar/lumbar curve was 62.3 ± 13.9° while the mean postoperative major curve angle was 20.8 ± 11.0°, with an average correction rate of 67.4 ± 13.4%.

**Table 2:** Comparison of Radiological Parameters between Age Groups

Radiological; mean ± SD	Age 10–18	Age 19–30	Total	Mean difference	***p***-value
**Major thoracic**					
n (%)	663 (62.7)	136 (12.9)	798 (75.6)		
Preoperative (°)	68.4 ± 18.2	65.4 ± 15.9	67.9 ± 17.8	3.0 ± 1.7	0.08
Postoperative (°)	23.6 ± 11.8	26.0 ± 13.2	24.0 ± 12.1	2.4 ± 1.1	0.04
Correction rate (%)	66.1 ± 11.6	61.6 ± 11.7	65.3 ± 11.7	4.5 ± 1.1	0.00
**Major thoracolumbar/ lumbar**					
n (%)	220 (20.8)	38 (3.6)	258 (24.4)		
Preoperative (°)	61.7 ± 12.9	65.5 ± 18.4	62.3 ± 13.9	3.8 ± 3.1	0.23
Postoperative (°)	19.8 ± 9.9	27.0 ± 14.9	20.8 ± 11.0	7.2 ± 2.5	0.01
Correction rate (%)	68.5 ± 13.1	60.9 ± 13.1	67.4 ± 13.4	7.6 ± 2.3	0.00

Values are expressed in mean ± SD

Perioperative parameters were described in Table 1. The mean duration of surgery was 146.8 ± 49.4 min. The average intraoperative blood loss was 952.9 ± 530.4 ml with 53 patients (5%) required allogeneic blood transfusion. Mean number of fusion levels per patient was 10.3 ± 2.2 levels. The average number of screws used in each patient was 14.1 ± 2.4 screws, with screw density of 1.3 ± 0.2 screws per fusion level. The average hospital stay after surgery was 3.5 ± 0.9 days. The mean Hb level at day 2 postoperatively was 10.9 ± 1.5 g/dl with a mean drop of Hb level of 2.8 ± 1.5 g/dl.

### Perioperative complications

A total of 24 perioperative complications was observed, giving an overall complication rate of 2.27%. 0.95% rate of major complications and 1.32% rate of minor complications were recorded. Prevalence of non-neurologic postoperative complications following corrective surgery was 1.80% with infection being the leading risk of complication at 1.32%. Superficial surgical site infection occurred in 12 patients (1.14%). Transient neurological deficit occurred in 5 patients (0.47%), followed by 2 cases (0.19%) of deep infection, and superior mesenteric artery syndrome. The remaining causes were singular events, which included, massive intraoperative blood loss, intraoperative seizure, and lung atelectasis (0.09% each) (Table 3). Within the perioperative period, we did not encounter any cases of proximal junctional kyphosis, implant failure and revision surgeries.

**Table 3:** Perioperative complications of AIS surgery based on Harms Study Group Complication Categories

Complications	Overall (***n*** = 1057)	%
Major		
In-hospital death	0	0
Transient neurological deficit		
Spinal Cord	$$ \left.\begin{array}{c}1\\ {}4\end{array}\right\}{\displaystyle \begin{array}{c}5\\ {}\end{array}} $$	$$ \left.\begin{array}{l}0.09\\ {}0.38\end{array}\right\}{\displaystyle \begin{array}{c}0.47\\ {}\end{array}} $$
Nerve Roots		
Deep infection	2	0.19
Superior mesenteric artery syndrome	2	0.19
Massive blood loss	1	0.09
Minor		
Superficial infection	12	1.14
Intraoperative seizure	1	0.09
Lung atelectasis	1	0.09
**Overall complication rate**	**24**	**2.27**

### Superficial SSI

**Fig. 1:**
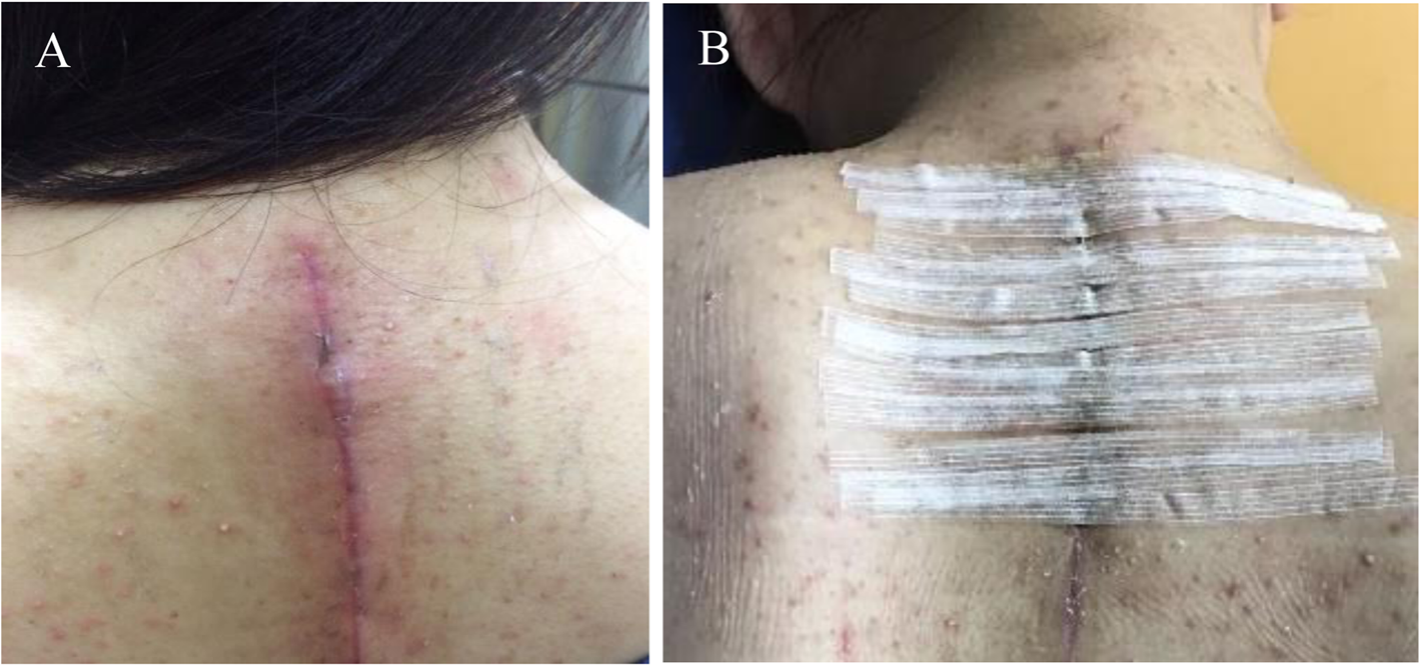
Superficial wound infection (**a**). The wound was approximated with adhesive strips (**b**)

All twelve patients presented with superficial infection had superficial wound breakdown at the proximal aspect of the wound. These patients underwent removal of subcuticular and subdermal sutures and followed by daily dressing for 5–7 days with antibiotics coverage. The wound was later approximated using adhesive strips. All wounds healed well without bringing the patients back to operation theatre. Wound culture was negative in all twelve patients (Fig. 1).

### Deep SSI

**Fig. 2:**
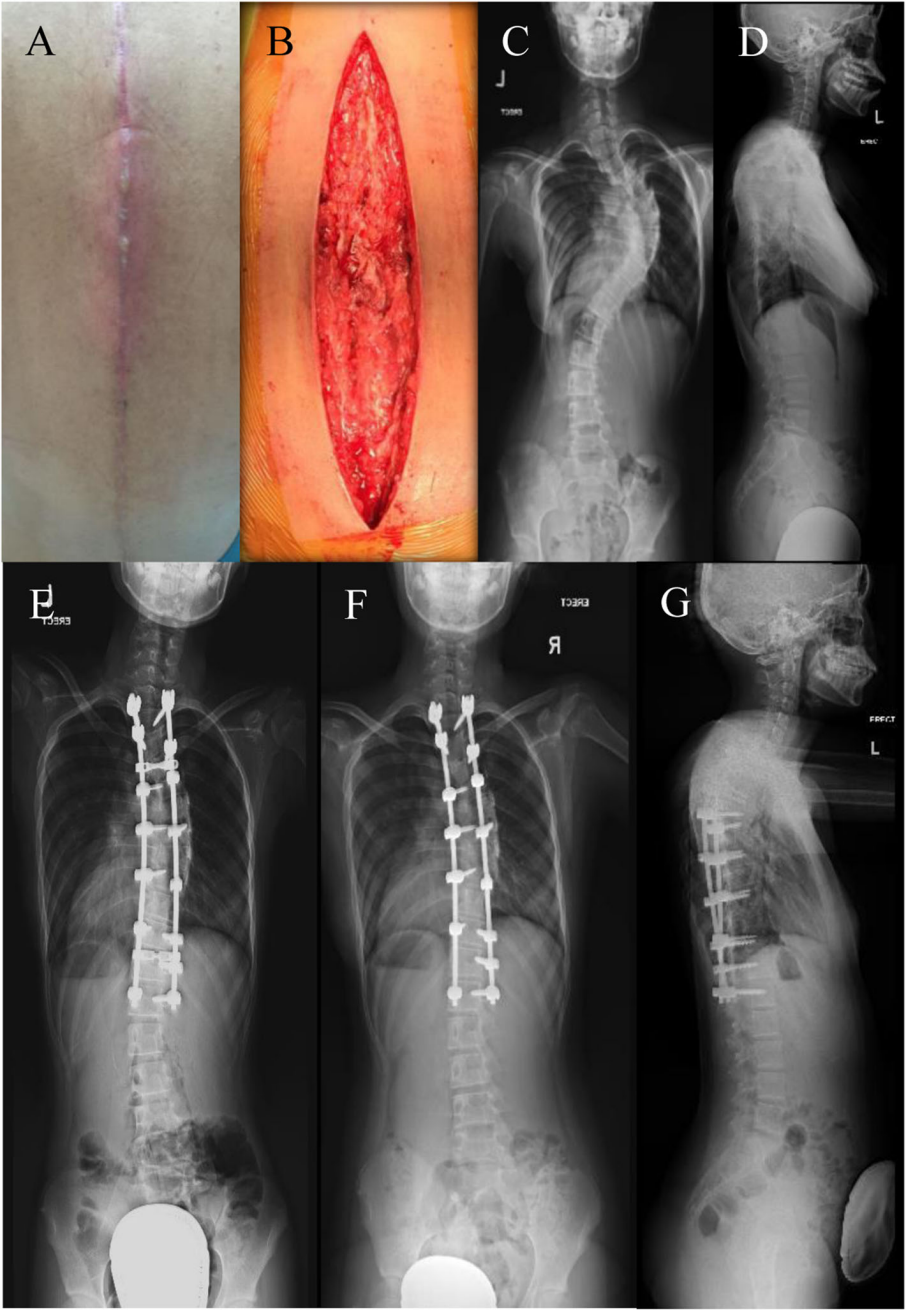
A patient developed deep surgical site infection 3 weeks postoperatively; **a** showed preoperative swelling and redness over surgical site; **b** was post-debridement intraoperatively). **c** & **d** were preoperative and **e** was postoperative AP radiograph. She was treated successfully with early debridement and removal of crosslinks within 24 h of presentation (**f** & **g**)

Two patients developed deep wound infection. One patient developed deep infection at day 27 post-operative and was treated successfully with early debridement with removal of crosslinks within 24 h of presentation. Culture was methicillin-sensitive *Staphylococcus aureus*. Intravenous Cefazolin and Gentamicin were administered for 4 weeks followed by oral Cefazolin for 5 months. Implant was successfully retained (Fig. 2). The second case of deep infection was a 12-year-old girl who had Lenke 1B curve with the Cobb angle of 57°. She underwent PSF from T3-T12. On postoperative Day 11, she presented with redness over the proximal aspect of the wound and had purulent discharge on postoperative Day 14. She underwent wound debridement and the implant was retained. The tissue culture grew Methicillin Resistant *Staphylococcus epidermidis* (MRSE). After wound debridement, she was given 3 days of IV vancomycin and 19 days of Tab Linezolid. Subsequently, she was covered with another 9 weeks of Tab Rifampicin and Tab Bactrim. Both the wounds were well-healed 2 years after the debridement.

### Transient neurological deficit

**Fig. 3:**
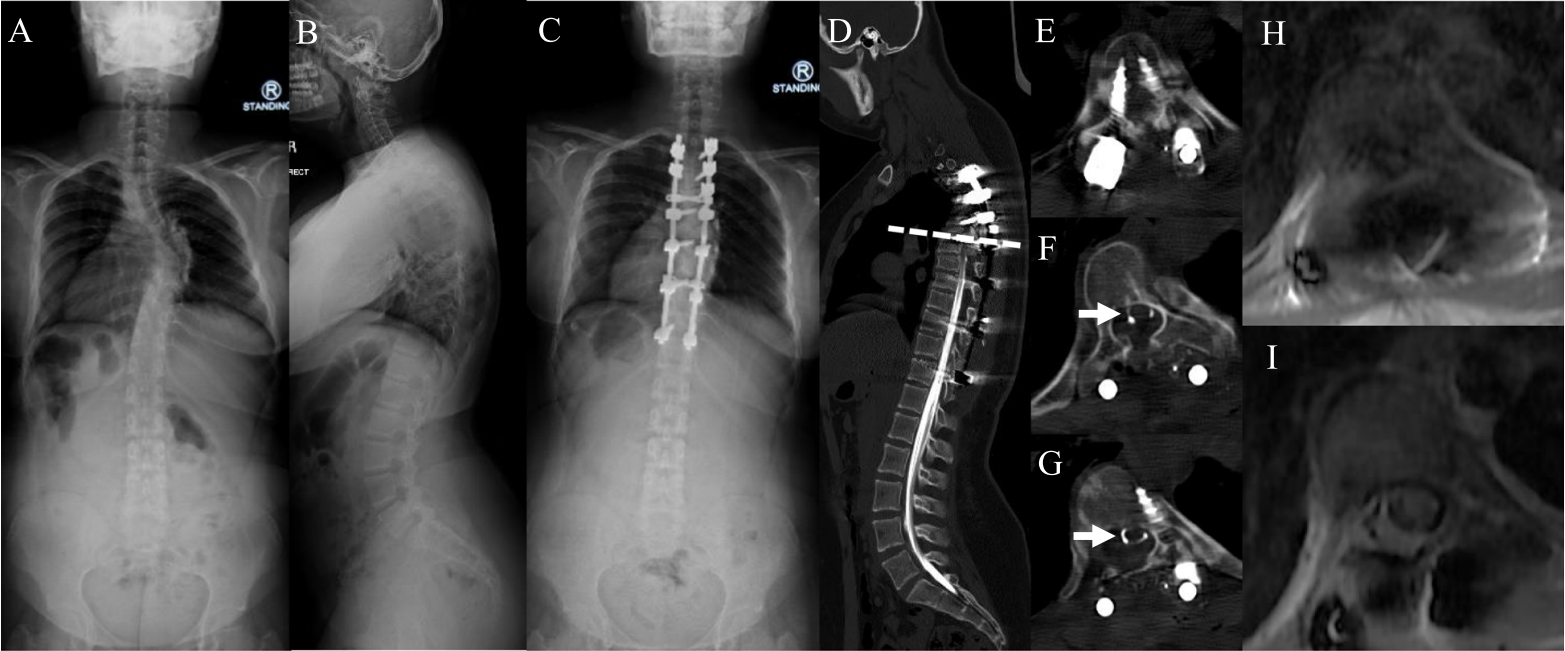
One patient (preoperative x-ray – **a** & **b**, postoperative AP x-ray – **c**) developed transient neurological deficit upon reversal of anesthesia. Urgent computed tomography (CT) myelography demonstrated cessation of contrast at T5 pedicle level in the sagittal view (dashed line in **d**) and axial view (**e**). Axial view at 5 mm (**f**) and 10 mm (**g**) below T5 level showed a thin layer of contrast within the thecal sac. Magnetic resonance imaging (MRI) showed epidural hematoma with spinal cord compression at T5/6 level (**h**). The spinal cord was visible at 15 mm below T5/6 level (**i**). Clinical assessment, MRI whole spine and CT thoracolumbosacral did not show any secondary causes for scoliosis

One patient developed transient neurological deficit upon reversal of anaesthesia. Intraoperative neuromonitoring was normal. Urgent computed tomography (CT) and magnetic resonance imaging (MRI) was suggestive of spinal epidural hematoma (SEH) at T5 level. Fortunately, she had spontaneous neurological recovery 3 h after surgery after completed the imaging studies. She regained a full ability to stand and walk and discharged well after 7 days (Fig. 3). There were four patients who developed postoperative radicular pain i.e. right L2, left L2, right T10 and right T5 dermatomes. These radicular pains lasted from 1 to 4 weeks duration and resolved with conservative management with the help of neuropathic medications.

### SMA syndrome

**Fig. 4:**
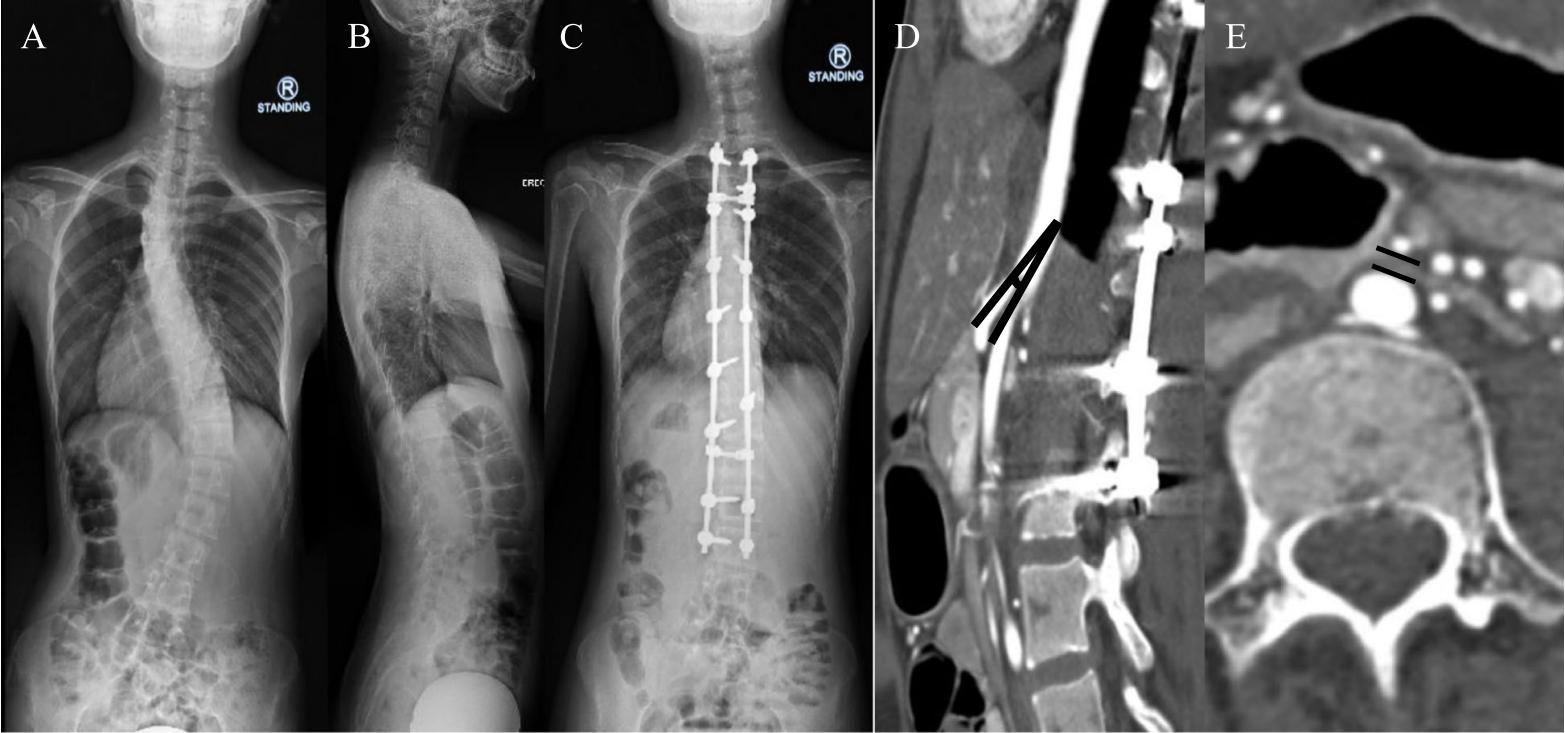
One case with superior mesenteric artery (SMA) syndrome after scoliosis surgery (preoperative x-ray – **a** & **b**, postoperative AP x-ray – **c**). Sagittal (**d**) and axial (**e**) of computed tomography angiography (CTA) of the abdomen revealed aortomesenteric angle of 10°, SMA-aorta distance of 3.2 mm and constriction of 3rd part of duodenum by the branches of the SMA

**Fig. 5:**
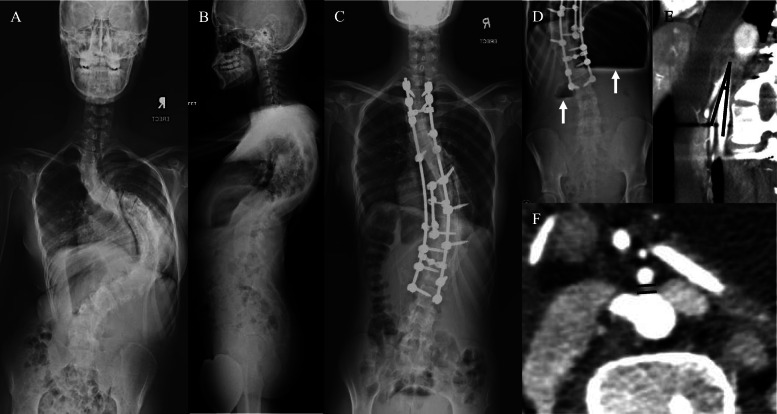
Another case with superior mesenteric artery (SMA) syndrome after scoliosis surgery (preoperative x-ray – **a** & **b**, postoperative AP x-ray – **c**) Erect abdominal radiograph (**d**) showed double bubble sign (white arrows). Sagittal (**e**) and axial (**f**) of CTA of the abdomen revealed an Aortomesenteric angle of 10° and SMA-aorta distance of 2.6 mm. Clinical assessment, MRI whole spine and CT thoracolumbosacral did not show any secondary causes for scoliosis

Two patients (one Lenke 6C and one Lenke 2AR curve), with Cobb angle of 49° and 132°, and low BMI of 16.4 kg/m^2^ and 16.3 kg/m^2^ respectively, presented with SMA syndrome following PSF T2-L3 with complaints of recurrent bouts of vomiting and weight loss. Abdominal radiographs showed distended gastric shadow. Computed Tomography Angiography (CTA) abdomen showed decreased Aortomesenteric angle and SMA-aorta distance. Principles of management involved gastric decompression with nasogastric tube, correction of electrolytes imbalance and nutritional support with low volume, high calorie nutritional supplement. Both patients were then started with small but frequent meals. Both patients were treated successfully with conservative management and discharged at day 24 and day 26 respectively (Figs. 4 & 5).

### Massive blood loss

One patient had massive intraoperative blood loss of 6.0 l. Blood was replaced with blood salvaged from the cell saver system. Additionally, patient received allogeneic blood transfusion. The patient required inotropic support during surgery and was admitted to intensive care unit postoperatively for 1 day.

### Intraoperative seizure

One patient with Lenke 6C curve developed generalized tonic-clonic seizure, which occurred 1 h into surgery, lasted for 1 min and aborted spontaneously. Postoperatively, there was no neurological deficit. MRI of the brain, intraoperative electrolytes and calcium levels were normal. No acidosis or hypothermia was documented.

### Lung atelectasis

**Fig. 6:**
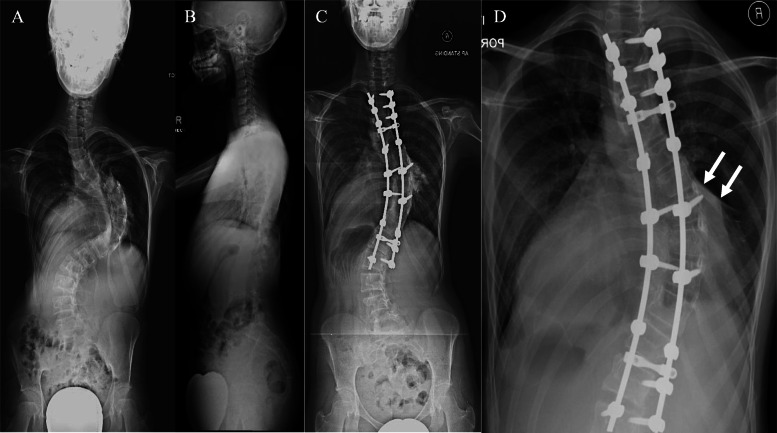
A 13-year-old girl with severe rigid scoliosis with preoperative Cobb angle of 117° (**a** & **b**) underwent PSF from T2 to L2. Postoperative Cobb angle was 60° and correction rate was 48.7% (**c**). She developed lung atelectasis of right middle and lower lobes at postoperative day 2 (**d**) which resolved at postoperative day 6 with non-invasive oxygen supplementation and chest physiotherapy

One patient developed lung atelectasis at day two postoperatively. Chest radiograph showed atelectasis of right middle and lower lobes. Her condition improved after intensive chest physiotherapy and oxygen supplementation and was discharged well at day six postoperatively (Fig. 6).

## Trend analysis

**Fig. 7:**
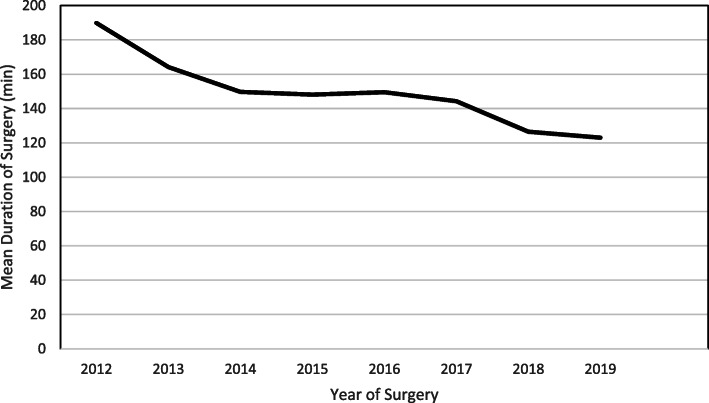
Graph of Mean Duration of Surgery by Year

**Fig. 8:**
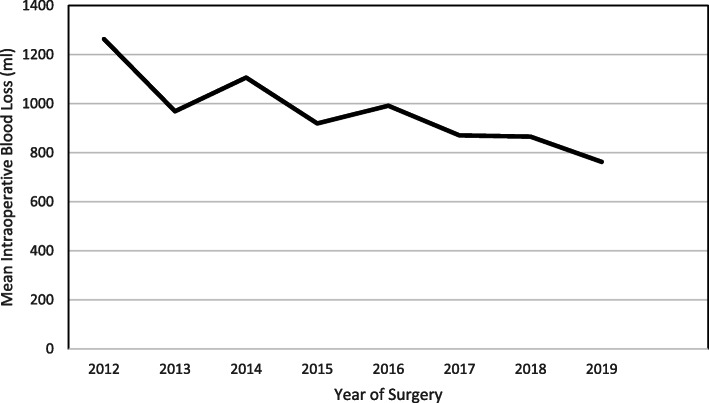
Graph of Mean Intraoperative Blood Loss by Year

Annual complication rate ranged from 0.88% (Year 2017) to 4.29% (Year 2016). The overall complication rate increased marginally from 1.89% (*n* = 475) during 2012 to 2015 to 2.57% (*n* = 582) during 2016 to 2019. One-way ANOVA analysis revealed insignificant change with regards to year-over-year (2012 till 2019) mean difference in terms of correction rate (*p* ≥ 0.05). In contrast, the mean duration of surgery improved throughout the study period (189.9 ± 60.9 min, year 2012 to 123.0 ± 39.3 min, year 2019) with a plateau between 2014 to 2017 (Fig. 7). The mean intraoperative blood loss, although fluctuated by year, also showed improvement from 1263.6 ± 621.6 ml for year 2012 to 762.3 ± 360.3 ml for year 2019 (Fig. 8). When we sub-analyzed between age groups (younger: 10–18 years old (*n* = 883) vs older: 19–30 years old (*n* = 174)), we found that the duration of surgery was higher in the older age group (mean difference: 17.2 ± 4.1 min, *p* < 0.001), whereas total blood loss and number of fusion levels were similar between the groups (mean difference: 45.1 ± 44.0 ml, *p* ≥ 0.05; 0.04 ± 0.2 level, p ≥ 0.05 respectively). Pre-operatively, there were no significant difference in curve angles between the age groups amongst those with major thoracic curve (mean difference: 3.0 ± 1.7°, p ≥ 0.05) and major thoracolumbar/lumbar curve (mean difference: 3.8 ± 3.1°, p ≥ 0.05). Post-operatively, correction rate was higher in the younger age group for both major thoracic (mean difference: 4.5 ± 1.1%, *p* < 0.01 and major thoracolumbar/lumbar curve groups (7.6 ± 2.3%, p < 0.01) Out of 24 reported complications, one patient in the older age group (19–30 years) had superficial infection.

## Discussion

The reported overall perioperative complication rate for AIS surgery ranged from 3.6 to 27.3% [2, 4, 10–12, 15, 16, 18, 31–34]. However, most literatures included both idiopathic and non-idiopathic scoliosis [2, 18, 35] utilizing different approaches (anterior, posterior or combined anterior-posterior) [4, 15–17]. The Scoliosis Research Society reported that overall complication rates were similar for anterior (5.2%) versus posterior approaches (5.1%) [11]. However, combined anterior-posterior procedures demonstrated twice as high (10.2%) complication rates [11]. Some authors believed that AIS patients that underwent combined procedures were at higher risk of complication as they tend to have more severe spinal curves [11] which predisposed to more comorbid conditions [16]. In our study, the overall complication rate of posterior approach in this cohort of AIS patients was 2.27% with major complication rate < 1%. The summary of the previous literatures which include only AIS patients who underwent scoliosis corrective surgery is illustrated in Table 4.

**Table 4:** Summary of published literatures on perioperative outcomes and complications of AIS surgery

Paper	Year	Single/ Multi	N	Approach	Operation Time (mins)	Blood Loss (ml)	Length of Stay (days)	Allogeneic Blood Transfusion	Complication Rate	Mortality	Neurologic Deficit	Infection	Respiratory	DVT/PE
Present paper	2021	S	1057	PSF	146.8 ± 49.4	952.9 ± 530.4	3.5 ± 0.9	53 (5)	24 (2.27)	0	5 (0.47)	14 (1.32)	1 (0.09)	0
Koo et al. [36]	2020	M	3759	PSF	*NA*	*NA*	5.16 ± 2.8	721(19.2)	864 (23.0)	*NS*	*NS*	51 (1.7)	60 (1.6)	0
Vavruch et al. [37]	2019	M	27	PSF	255 ± 48	1615 ± 901	8 ± 1.3	*NA*	7 (25.9)	0	*NS*	2 (7.4)	0	*NS*
Bartley et al. [28]	2017	M	3582	ASF, PSF and APSF	*NA*	*NA*	NA	*NA*	93 (2.6)	1 (0.03)	19 (0.53)	20 (0.56)	13 (0.36)	0
Menger et al. [12]	2017	M	75,106	*NS*	*NA*	*NA*	5.72	*NA*	6451 (8.59)	69 (0.1)	648 (0.9)	496 (0.4)	2068 (2.8)	73 (0.1)
Sugawara et al. [10]	2017	M	458	*NS*	*NA*	*NA*	*NA*	*NA*	37 (8.08)	0	12 (2.62)	11 (2.4)	1 (0.22)	1 (0.22)
de Bodman et al. [38]	2017	S	70	MIS PSF	337.1 ± 121.3	345.7 ± 175.1	4.6 ± 0.8	0	8 (11.4)	0	0	4 (5.7)	1 (1.4)	1 (1.4)
Chiu et al. [39]	2016	S	100	PSF	188.5 ± 53.4	951 ± 454	3.5 ± 0.9	*NA*	*NA*	*NA*	*NA*	*NA*	*NA*	*NA*
Vigneswaran et al. [16]	2015	M	1783–5228	ASF, PSF and APSF	*NA*	*NA*	5.6 ± 5.7 – 6.6 ± 6.2	*NA*	279 (15.65)– 1167 (22.32)	1 (0.02) – 4 (0.22)	< 1%	43 (2.4) – 89 (1.7)	< 1%	< 1%
Vigneswaran et al. [16]	2015	M	1130–4917	PSF only	*NA*	*NA*	5.4 ± 5–5.9 ± 4.2	*NA*	164 (14.5) – 1010 (27.3)	9 (0.06)	< 1%	50 (4.4) – 98 (2)	< 1%	< 1%
Basques et al. [33]	2015	M	733	PSF	> 365 mins in 108 (14.7%)	*NA*	> 6 days in 60 (8.2%)	*NA*	27 (3.7)	0	3 (0.4)	15 (2.0)	1 (0.1)	0
Imajo et al. [40]	2015	M	1485	ASF, PSF and APSF	*NA*	*NA*	*NA*	*NA*	245 (16.5)	1 (0.07)	31 (2.1)	*NS*	*NS*	*NS*
Tarrant et al. [14]	2014	M	77	PSF	330.0^a^ (258.0–420.0)	1012 (791–1400)	10^a^ (8–11)	36 (46.8)	4 (5.2)	0	*NA*	5 (6.5)	19 (24.68)	0
Cristante et al. [35]	2014	S	94	PSF	329.99 (307.95–358.02)	*NA*	*NA*	43 (47.7)	*NA*	*NA*	*NA*	*NA*	*NA*	*NA*
Roberts at al [13]	2014	S	72	PSF	139.0 (108–190)	419 (180–750)	6.1 (5–8)	*NA*	1 (1.4)	0	1 (1.4)	0	0	0
Divecha et al. [18]	2014	M	9295	NS	*NA*	*NA*	*NA*	*NA*	339 (3.64)	17 (0.19)	100 (1.1)	222 (2.4)	*NA*	*NA*
Yilmaz et al. [25]	2012	S	35	PSF	351.5 ± 73.2	1211.7 ± 643.7	NA	*NA*	1 (2.85)	NA	0	1 (2.85)	0	0
Fu et al. [31]	2011	M	23,918	*NS*	*NA*	*NA*	NA	*NA*	2040 (8.5)	31 (0.13)	324 (1.4)	661 (2.7)	224 (0.9)	17 (0.07)
Reames et al. [2]	2011	M	11,227	*NS*	*NA*	*NA*	NA	*NA*	760 (6.3)	2 (0.02)	86 (0.8)	156 (1.4)	63 (0.6)	6 (0.05)
Hod-Feins et al. [9]	2007	S	95	ASF, PSF and APSF	402	*NA*	3.23	*NA*	*NA*	0	2 (2.1)	0	5 (5.2)	0
Carreon et al. [4]	2007	M	702	ASF, PSF and APSF	*NA*	*NA*	*NA*	*NA*	108 (15.4)	0	*NS*	5 (0.71)	13 (1.85)	0
Coe et al. [11]	2006	M	4369	PSF	*NA*	*NA*	NA	*NA*	221 (5.05)	2 (0.05)	14 (0.32)	59 (1.35)	42 (0.96)	3 (0.07)

Figures are expressed in means (range) or ± SD, unless specified. ^a^Figures are expressed in median. *NA* Not available, *NS* Not specified, *ASF* Anterior spinal fusion, *PSF* Posterior spinal fusion, *APSF* Anterior and posterior spinal fusion, *S* Single-center, *M* Multi-center, *DVT* Deep vein thrombosis, *PE* Pulmonary embolism

Wound complications carry significant morbidity to patients and increase health-care costs because of longer hospital stay, surgical debridement, and washout procedures, and the potential need for removal of spinal implants [41]. Direct comparisons are difficult to make due to wide variations in age groups, surgical approaches and aetiologies reported in many studies. SSI rates from the SRS Morbidity and Mortality database series varied between 0.17 and 1.37% [1, 2, 11]. Most recent and largest multi-centre analysis involving 84,320 cases of AIS surgeries in the SRS database reported an overall SSI rate of 0.52% [1]. Tipper et al. [42] proved that adoption of a standardized protocol resulted in significant reduction of SSI risk in pediatric scoliosis surgery.

Intraoperative blood loss in our series was comparable with other studies, which ranged from 822 to 1212 ml [4, 7, 15, 17, 25, 39], with a reported 5% incidence of allogeneic blood transfusion. Although our mean duration of surgery did not differ much year-over-year despite the involvement of different assistants throughout the study period, the volume of blood loss per level fused had shown a slow progressive decline from 2012 to 2019. Operative time is a major factor that correlated with estimated blood loss. However, there were other subjective factors that could not be measured such as the amount of effort taken to secure haemostasis (from soft tissue and from the bone). These could have a significant effect on the intraoperative blood loss independent of the operative time.

Preoperatively, patients’ haemoglobin were optimized to ensure better physiologic reserve (mean preoperative haemoglobin of 13.7 ± 1.2 g/dl). As part of the accelerated recovery protocol, patients were prescribed oral Iberet Folic 500 (multivitamins and minerals) 1 tablet once a day 1 month prior to operation [30]. Multivariate analysis by Liang et al. [43] in 110 operated scoliosis patients demonstrated that higher preoperative haemoglobin (OR: 0.901, *p* = 0.001) predicted lower allogeneic intraoperative transfusion rates. In their study, mean preoperative haemoglobin values were higher in patients without transfusion than in those with transfusion (13.70 g/dL versus 12.75 g/dL, *p* = 0.000). Fernandez et al. [44] also reported lower probability of transfusion with higher preoperative haemoglobin. Apart from that, usage of cell salvage system resulted in reduced need for blood transfusion [45]. Our study reported mean post-operative haemoglobin level of 10.9 ± 1.5 g/dl with a mean drop of haemoglobin of 2.8 ± 1.5 g/dl and an average 51.4% return of EBL. This coincides with a study by Bowen et al. [46] who reported an average of 53% return of EBL as cell salvage system transfusion with a greater than 5-fold reduction in the allogeneic transfusion rate in the cell salvage system group. Preoperative optimisation of patients’ haemoglobin and nutritional status and usage of cell saver, along with other blood conservation strategies, help obviate the need for blood transfusion in our patient population. Usage of prophylactic intravenous tranexamic acid also reduce blood loss [47, 48] and its possible complications.

In addition, an accelerated recovery protocol following PSF for AIS patients was adopted in our centre. It comprised of preoperative regime, preoperative day of surgery counselling, intraoperative strategies to shorten the duration of operation and to minimize blood loss and an accelerated postoperative rehabilitation and pain management strategies [30]. Adoption of this accelerated recovery protocol had led to a short average length of hospital stay of 3.5 ± 0.9 days in our patients. Recently published data on the development and implementation of standardized care pathways showed that these pathways are effective in lowering postoperative pain scores, perioperative transfusion requirement and postoperative length of stay without an increase in postoperative complications [49–51].

The mean operation time of our study was 146.8 ± 49.4 min with mean total fused level of 10.3 levels. This was comparatively lower than previous reports which ranged from 139 to 365 min [4, 25, 33]. Basques et al. [33], in his retrospective database review of 733 AIS patients who underwent PSF, reported a higher average operative time of 275 ± 90 min, with 108 patients (14.7%) having had operative time exceeding 365 min. However, the longer operative time may be influenced by variability in surgical technique and number of procedures performed as it is multicentre study involving different surgeons and institutions. Yilmaz and colleagues [25], in a comparative study of 105 AIS patients who underwent posterior corrective surgery, reported a significantly longer mean operation time of 5.85 h among the pedicle screw group (screw density: 1.4 ± 0.24) than the other groups (hook and hybrid construct). The authors cited that these cases were performed early in the authors’ experience with thoracic pedicle screws. Roberts et al. [13] reported a mean operation time of 139 min in a retrospective cohort of 72 patients. However, the patient population was of Lenke type-5C thoracolumbar/lumbar AIS subtype that was treated by PSF using a unilateral convex segmental pedicle screw technique which required lower number of fused segment (mean: 6.2 levels fused). Carreon et al. [4] found that prolonged posterior surgery time, total anaesthesia time, and increased operative blood loss were associated with higher rates of non-neurologic complications. The authors determined that a cut-point of 775 ml of blood loss and 368 min of total anaesthesia time best discriminates between patients with and without complications.

Wide heterogeneity in implant density exists amongst surgeons in AIS surgery with mean implant density varying from 1.06 to 2.0 implants per level fused which can achieve mean percentage of major curve angle correction from 64 to 70% [52]. Our implant density (mean: 1.27 ± 0.21) was considered low as defined by various published studies [53–55]. With this implant density, we had achieved correction rates of 65.3 ± 11.7% for thoracic curves and 67.4 ± 13.4% for thoracolumbar/lumbar curves. In addition, no posterior column osteotomies were performed, and spinal mobilization was achieved only with facetectomy. We believed that the relatively low implant density and obviating the need for posterior column osteotomies in this cohort may account for shorter operation time and lesser intraoperative blood loss.

Published literature showed significant reduction in operative time [56, 57] and blood loss [53, 56] in low-density group (LD) group. Shen et al. [56] reviewed 62 consecutive Lenke 1 AIS patients who underwent posterior spinal arthrodesis using all-pedicle screw instrumentation and found that LD group (< 1.61 screws/level) reported shorter operating time (278.4 vs. 331.0 min, *p* = 0.004) and lower blood loss (823.6 vs. 1010.9 ml, *p* = 0.048). Kemppainen et al. [57] reviewed 52 AIS patients and found that LD group not only achieved comparable sagittal alignment, coronal balance, and translation of the major apical vertebra when compared with HD group, but also reduced operative time (267 vs. 309 min, *p* < 0.01) and estimated direct cost ($16,126 vs. $21,967, p < 0.01). The authors found significantly less blood reinfused by the cell saver in the LD group (254 vs. 352 mL, *p* = 0.042) using volume of reinfused cell saver blood as a more accurate surrogate for blood loss. Since low-density constructs are shown to be safe and clinically equivalent to higher density constructs, fewer pedicle screws could be used, which will reduce cost of hospitalization [55], operative time, blood loss, radiation and risk of screw malposition [52].

There were several limitations in our study. The study was designed to report on the perioperative outcome for AIS patients. As such, long-term results (loss of correction) and late complication rates (delayed infections, non-unions, instrumentation failure, crankshaft phenomenon, etc.) were not reported in this study. The different Lenke types, flexibility of the curves and skeletal maturity of patients may influence surgical strategy and level selection for fusion. This may affect the outcomes and potential bias may occur if there is a need to infer to certain subgroups. This study categorized the major and minor complications based on complication categories as proposed by Harms Study Group [28] and therefore is only comparable with other studies which uses this classification. This study spanned over 8 years duration. Over this duration, there may be variations in surgical strategy and technique that could influence the outcome. Blood loss recorded was best estimates with unaccountable blood loss on patients’ drapes, surgeon gloves, and instruments. We did not assess screw accuracy on postoperative radiographs and did not analyse the changes of screw accuracy over the years. We did not perform a comparison between low-density (LD) construct and high-density (HD) construct in our retrospective study as our mean implant density was largely low-density at 1.27 ± 0.21 screws/level. We did not assess the postoperative restoration of kyphosis and rotational correction of patients. In our institution, we did not perform immediate post-op lateral view. The reason, we would like to reduce unnecessary radiation since the patient has already had many radiographs taken prior to surgery. Our first postoperative lateral view radiograph will be taken at 8–12 weeks follow up. Previous studies have been inconsistent in demonstrating effect of screw density (low-density versus high-density) on postoperative kyphosis [58, 59]. Future studies are warranted to further explore these issues.

## Conclusion

AIS patients treated with single-staged PSF in this cohort using alternate pedicle screw construct with low implant density demonstrated an optimum correction rate of 65.3% for thoracic curves and 67.4% for thoracolumbar/lumbar curves, with 0.95% rate of major complications and 1.32% rate of minor complications. The prevalence of non-neurologic postoperative complications following corrective surgery was 1.80% with infection being the leading risk of complication at 1.32%. These findings should guide surgeons in their preoperative counselling with patients and practice vigilance in wound follow-up. Alternate level pedicle-screw placement after the creation of stable proximal and distal foundations leads to lower implant density and its associated lower cost of surgery without sacrificing safety.

## Data Availability

The datasets used and analysed during the current study are available from the corresponding author upon reasonable request.

## References

[1] Kwan KYH, Koh HY, Blanke KM, Cheung KMC (2020). Complications following surgery for adolescent idiopathic scoliosis over a 13-year period. Bone Joint J.

[2] Reames DL, Smith JS, Fu KM, Polly DW Jr, Ames CP, Berven SH, Perra JH, Glassman SD, McCarthy R, Knapp RD Jr, Heary R, Shaffrey CI, Scoliosis Research Society Morbidity and Mortality Committee (2011). Complications in the surgical treatment of 19,360 cases of pediatric scoliosis: a review of the Scoliosis Research Society morbidity and mortality database. Spine (Phila Pa 1976).

[3] Burton DC, Carlson BB, Place HM, Fuller JE, Blanke K, Cho R, Fu KM, Ganju A, Heary R, Herrera-Soto JA, Larson AN, Lavelle WF, Nelson IW, Vernengo-Lezica A, Verska JM (2016). Results of the Scoliosis Research Society morbidity and mortality database 2009-2012: a report from the morbidity and mortality committee. Spine Deform..

[4] Carreon LY, Puno RM, Lenke LG, Richards BS, Sucato DJ, Emans JB, Erickson MA (2007). Non-neurologic complications following surgery for adolescent idiopathic scoliosis. J Bone Joint Surg Am.

[5] Kuklo TR, Owens BD, Polly DW (2003). Perioperative blood and blood product management for spinal deformity surgery. Spine J.

[6] Yu X, Xiao H, Wang R, Huang Y (2013). Prediction of massive blood loss in scoliosis surgery from preoperative variables. Spine (Phila Pa 1976).

[7] Koerner JD, Patel A, Zhao C, Schoenberg C, Mishra A, Vives MJ, Sabharwal S (2014). Blood loss during posterior spinal fusion for adolescent idiopathic scoliosis. Spine (Phila Pa 1976).

[8] Christodoulou AG, Givissis P, Symeonidis PD, Karataglis D, Pournaras J (2006). Reduction of postoperative spinal infections based on an etiologic protocol. Clin Orthop Relat Res.

[9] Hod-Feins R, Abu-Kishk I, Eshel G, Barr Y, Anekstein Y, Mirovsky Y (2007). Risk factors affecting the immediate postoperative course in pediatric scoliosis surgery. Spine (Phila Pa 1976).

[10] Sugawara R, Takeshita K, Arai Y, Takaso M, Takahashi J, Hosoe H, Doi T, Shimizu K (2017). Morbidity & Mortality Survey of spinal deformity surgery in 2012-report by the Japanese scoliosis society. Spine Surg Related Res.

[11] Coe JD, Arlet V, Donaldson W (2006). Complications in spinal fusion for adolescent idiopathic scoliosis in the new millennium. A report of the Scoliosis Research Society Morbidity and Mortality Committee. Spine (Phila Pa 1976).

[12] Menger RP, Kalakoti P, Pugely AJ, Nanda A, Sin A (2017). Adolescent idiopathic scoliosis: risk factors for complications and the effect of hospital volume on outcomes. Neurosurg Focus.

[13] Roberts SB, Tsirikos AI, Subramanian AS (2014). Posterior spinal fusion for adolescent idiopathic thoracolumbar/lumbar scoliosis: clinical outcomes and predictive radiological factors for extension of fusion distal to caudal end vertebra. Bone Joint J.

[14] Tarrant RC, O'Loughlin PF, Lynch S (2014). Timing and predictors of return to short-term functional activity in adolescent idiopathic scoliosis after posterior spinal fusion: a prospective study. Spine (Phila Pa 1976).

[15] Betz RR, Harms J, Clements DH (1999). Comparison of anterior and posterior instrumentation for correction of adolescent thoracic idiopathic scoliosis. Spine (Phila Pa 1976).

[16] Vigneswaran HT, Grabel ZJ, Eberson CP, Palumbo MA, Daniels AH (2015). Surgical treatment of adolescent idiopathic scoliosis in the United States from 1997 to 2012: an analysis of 20,346 patients. J Neurosurg Pediatr.

[17] Diab M, Smith AR, Kuklo TR, Spinal Deformity Study Group (2007). Neural complications in the surgical treatment of adolescent idiopathic scoliosis. Spine (Phila Pa 1976).

[18] Divecha HM, Siddique I, Breakwell LM (2014). Complications in spinal deformity surgery in the United Kingdom: 5-year results of the annual British scoliosis society National Audit of morbidity and mortality. Eur Spine J.

[19] Maruyama T, Takeshita K (2008). Surgical treatment of scoliosis: a review of techniques currently applied. Scoliosis..

[20] Weiss HR, Goodall D (2008). Rate of complications in scoliosis surgery - a systematic review of the pub med literature. Scoliosis..

[21] Bennett JT, Hoashi JS, Ames RJ, Kimball JS, Pahys JM, Samdani AF (2013). The posterior pedicle screw construct: 5-year results for thoracolumbar and lumbar curves. J Neurosurg Spine..

[22] Chan CYW, Mohamad SM, Tan SH, Loh LH, Lim JN, Chiu CK, Hasan MS, Kwan MK (2019). Do overweight adolescent idiopathic scoliosis (AIS) patients have an increased perioperative risk for posterior spinal fusion (PSF) surgery?: a propensity score matching analysis of 374 AIS patients. Spine (Phila Pa 1976).

[23] Hee HT, Yu ZR, Wong HK (2007). Comparison of segmental pedicle screw instrumentation versus anterior instrumentation in adolescent idiopathic thoracolumbar and lumbar scoliosis. Spine (Phila Pa 1976).

[24] Lehman RA, Lenke LG, Keeler KA, Kim YJ, Buchowski JM, Cheh G, Kuhns CA, Bridwell KH (2008). Operative treatment of adolescent idiopathic scoliosis with posterior pedicle screw-only constructs: minimum three-year follow-up of one hundred fourteen cases. Spine (Phila Pa 1976).

[25] Yilmaz G, Borkhuu B, Dhawale AA, Oto M, Littleton AG, Mason DE, Gabos PG, Shah SA (2012). Comparative analysis of hook, hybrid, and pedicle screw instrumentation in the posterior treatment of adolescent idiopathic scoliosis. J Pediatr Orthop.

[26] Lenke LG, Betz RR, Harms J, Bridwell KH, Clements DH, Lowe TG, Blanke K (2001). Adolescent idiopathic scoliosis: a new classification to determine extent of spinal arthrodesis. J Bone Joint Surg Am.

[27] Nadler SB, Hidalgo JH (1962). Bloch T prediction of blood volume in normal human adults. Surgery..

[28] Bartley CE, Yaszay B, Bastrom TP, Shah SA, Lonner BS, Asghar J, Miyanji F, Samdani A, Newton PO (2017). Perioperative and delayed major complications following surgical treatment of adolescent idiopathic scoliosis. J Bone Joint Surg Am.

[29] Choo QQ, Chiu CK, Lisitha KA, Chan CYW, Kwan MK (2019). Quantitative analysis of local bone graft harvested from the posterior elements during posterior spinal fusion in adolescent idiopathic scoliosis patients. J Orthop.

[30] Chan CYW, Loo SF, Ong JY, Lisitha KA, Hasan MS, Lee CK, Chiu CK, Kwan MK (2017). Feasibility and outcome of an accelerated recovery protocol in Asian adolescent idiopathic scoliosis patients. Spine (Phila Pa 1976).

[31] Fu K-MG, Smith JS, Polly DW, Ames CP, Berven SH, Perra JH, Glassman SD, McCarthy RE, Knapp DR, Shaffrey CI, Scoliosis Research Society Morbidity and Mortality Committee (2011). Morbidity and mortality associated with spinal surgery in children: a review of the Scoliosis Research Society morbidity and mortality database. J Neurosurg Pediatr.

[32] Asher MA, Burton DC (2006). Adolescent idiopathic scoliosis: natural history and long term treatment effects. Scoliosis..

[33] Basques BA, Bohl DD, Golinvaux NS, Smith BG, Grauer JN (2015). Patient factors are associated with poor short-term outcomes after posterior fusion for adolescent idiopathic scoliosis. Clin Orthop Relat Res.

[34] Rihn JA, Lee JY, Ward WT (2008). Infection after the surgical treatment of adolescent idiopathic scoliosis: evaluation of the diagnosis, treatment, and impact on clinical outcomes. Spine (Phila Pa 1976).

[35] Cristante AF, Borges PA, Barbosa AR, Letaif OB, Marcon RM, Barros-Filho TE (2014). Predictive factors for perioperative blood transfusion in surgeries for correction of idiopathic, neuromuscular or congenital scoliosis. Clinics (Sao Paulo).

[36] Koo AB, Elsamadicy AA, Kundishora AJ, David WB, Lee M, Hong CS, Lee V, Kahle KT, DiLuna M (2020). Geographic variation in outcomes and costs after spinal fusion for adolescent idiopathic scoliosis. World Neurosurg.

[37] Vavruch L, Brink RC, Malmqvist M, Schlösser TPC, van Stralen M, Abul-Kasim K, Ohlin A, Castelein RM, Tropp H (2019). Surgical outcomes of anterior versus posterior fusion in Lenke type 1 adolescent idiopathic scoliosis. Spine (Phila Pa 1976).

[38] de Bodman C, Miyanji F, Borner B (2017). Minimally invasive surgery for adolescent idiopathic scoliosis: correction of deformity and peri-operative morbidity in 70 consecutive patients. Bone Joint J.

[39] Chiu CK, Chan CY, Aziz I (2016). Assessment of intraoperative blood loss at different surgical stages during posterior spinal fusion surgery in the treatment of adolescent idiopathic scoliosis. Spine (Phila Pa 1976).

[40] Imajo Y, Taguchi T, Yone K, Okawa A, Otani K, Ogata T, Ozawa H, Shimada Y, Neo M, Iguchi T (2015). Japanese 2011 nationwide survey on complications from spine surgery. J Orthop Sci.

[41] Mackenzie WG, Matsumoto H, Williams BA (2013). Surgical site infection following spinal instrumentation for scoliosis: a multicenter analysis of rates, risk factors, and pathogens. J Bone Joint Surg Am.

[42] Tipper GA, Chiwera L, Lucas J (2019). Reducing surgical site infection in pediatric scoliosis surgery: a multidisciplinary improvement program and prospective 4-year audit. Global Spine J.

[43] Liang J, Shen J, Chua S, Fan Y, Zhai J, Feng B, Cai S, Li Z, Xue X (2015). Does intraoperative cell salvage system effectively decrease the need for allogeneic transfusions in scoliotic patients undergoing posterior spinal fusion? A prospective randomized study. Eur Spine J.

[44] Fernandes P, Soares do Brito J, Flores I (2020). Blood Management and Risk Assessment for Transfusion in Pediatric Spinal Deformity Surgery. Adv Hematol.

[45] Mirza AH, Aldlyami E, Bhimarasetty C, Thompson AG, Spilsbury J, Marks DS (2009). The role of peri-operative cell salvage in instrumented anterior correction of thoracolumbar scoliosis: a case-controlled study. Acta Orthop Belg.

[46] Bowen RE, Gardner S, Scaduto AA, Eagan M, Beckstead J (2010). Efficacy of intraoperative cell salvage systems in pediatric idiopathic scoliosis patients undergoing posterior spinal fusion with segmental spinal instrumentation. Spine (Phila Pa 1976).

[47] Grant JA, Howard J, Luntley J, Harder J, Aleissa S, Parsons D (2009). Perioperative blood transfusion requirements in pediatric scoliosis surgery: the efficacy of tranexamic acid. J Pediatr Orthop.

[48] Neilipovitz DT, Murto K, Hall L, Barrowman NJ, Splinter WM (2001). A randomized trial of tranexamic acid to reduce blood transfusion for scoliosis surgery. Anesth Analg.

[49] Fletcher ND, Andras LM, Lazarus DE, Owen RJ, Geddes BJ, Cao J, Skaggs DL, Oswald TS, Bruce RW (2017). Use of a novel pathway for early discharge was associated with a 48% shorter length of stay after posterior spinal fusion for adolescent idiopathic scoliosis. J Pediatr Orthop.

[50] Gornitzky AL, Flynn JM, Muhly WT, Sankar WN (2016). A rapid recovery pathway for adolescent idiopathic scoliosis that improves pain control and reduces time to inpatient recovery after posterior spinal fusion. Spine Deform..

[51] Oetgen ME, Martin BD, Gordish-Dressman H, Cronin J, Pestieau SR (2018). Effectiveness and sustainability of a standardized care pathway developed with use of lean process mapping for the treatment of patients undergoing posterior spinal fusion for adolescent idiopathic scoliosis. J Bone Joint Surg Am.

[52] Larson AN, Aubin CE, Polly DW (2013). Are more screws better? A systematic review of anchor density and curve correction in adolescent idiopathic scoliosis. Spine Deform.

[53] Charalampidis A, Moller A, Wretling ML (2018). Implant density is not related to patient-reported outcome in the surgical treatment of patients with idiopathic scoliosis. Bone Joint J.

[54] Polly DW, Larson AN, Sponseller PD (2019). Prospective randomized controlled trial of implant density in AIS: results of the Minimize Implants Maximize Outcomes study. Spine J.

[55] Larson AN, Polly DW, Ackerman SJ (2016). What would be the annual cost savings if fewer screws were used in adolescent idiopathic scoliosis treatment in the US?. J Neurosurg Spine.

[56] Shen M, Jiang H, Luo M, Wang W, Li N, Wang L, Xia L (2017). Comparison of low density and high density pedicle screw instrumentation in Lenke 1 adolescent idiopathic scoliosis. BMC Musculoskelet Disord.

[57] Kemppainen JW, Morscher MA, Gothard MD, Adamczyk MJ, Ritzman TF (2016). Evaluation of limited screw density pedicle screw constructs in posterior fusions for adolescent idiopathic scoliosis. Spine Deform..

[58] Dial BL, Esposito VR, Catanzano AA, Fitch RD, Lark RK (2020). Implant Distribution Versus Implant Density in Lenke Type 1 Adolescent Idiopathic Scoliosis: Does the Position of the Screw Matter?. Global Spine J.

[59] Sudo H, Abe Y, Kokabu T, Ito M, Abumi K, Ito YM, Iwasaki N (2016). Correlation analysis between change in thoracic kyphosis and multilevel facetectomy and screw density in main thoracic adolescent idiopathic scoliosis surgery. Spine J.

